# Glycodendrimers: tools to explore multivalent galectin-1 interactions

**DOI:** 10.3762/bjoc.11.84

**Published:** 2015-05-12

**Authors:** Jonathan M Cousin, Mary J Cloninger

**Affiliations:** 1 Department of Chemistry and Biochemistry, Montana State University, Bozeman, MT 59717, USA

**Keywords:** dendrimer, galectin-1, glycodendrimer, multivalent, nanoparticle

## Abstract

Four generations of lactose-functionalized polyamidoamine (PAMAM) were employed to further the understanding of multivalent galectin-1 mediated interactions. Dynamic light scattering and fluorescence microscopy were used to study the multivalent interaction of galectin-1 with the glycodendrimers in solution, and glycodendrimers were observed to organize galectin-1 into nanoparticles. In the presence of a large excess of galectin-1, glycodendrimers nucleated galectin-1 into nanoparticles that were remarkably homologous in size (400–500 nm). To understand augmentation of oncologic cellular aggregation by galectin-1, glycodendrimers were used in cell-based assays with human prostate carcinoma cells (DU145). The results revealed that glycodendrimers provided competitive binding sites for galectin-1, which diverted galectin-1 from its typical function in cellular aggregation of DU145 cells.

## Introduction

**Figure 1:**
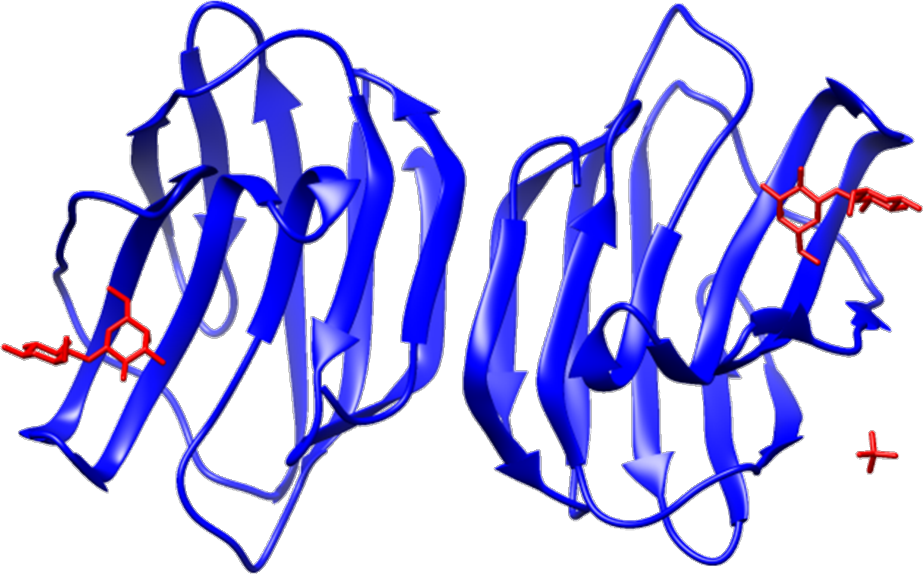
The structure of galectin-1. Reproduced with permission from [21]. Copyright 2004 Elsevier.

Galectin-1 is a multivalent protein that mediates biological activity through multivalent interactions with cell surface glycoconjugates [1–4]. Galectin-1 is a non-covalent homodimer that belongs to a family of β-galactoside binding proteins called galectins [5–7]. The monomeric units are oriented such that the two carbohydrate recognition domains are located on apposing faces of the dimer (Figure 1). Although individual binding interactions with carbohydrates are weak [8], ligands for galectin-1 typically possess an array of carbohydrates to enhance the binding affinity [1,9–10]. Galectin-1 binding to carbohydrates cross-links adjacent glycoconjugates to mediate biological activity [10–16]. Specifically, galectin-1 has been reported to be involved in multivalent mechanisms that cluster cell surface glycoproteins [10,17], cross-link receptors [13,18], and form lattices and larger aggregates [12,19–20]. Synthetic multivalent ligands displaying multiple copies of recognition elements are a logical tool to study mechanisms of galectin-1 mediated biological activities.

Mutivalent frameworks have been used to organize lectins and to mediate biological activity for the advancement of mechanistic understandings [22–25]. Synthetic multivalent ligands have been observed to enhance galectin-1 binding through the glycoside cluster effect by mediating the formation of cross-linked aggregates [26–28]. Tinari et al. observed galectin-1 augmentation of homotypic cellular aggregation in human melanoma cells (A375) through bivalent binding of 90K/Mac-2BP, a cell surface glycoprotein [29]. To further the understanding of structural specificity in binding events, Iurisci et al. designed multivalent oligosaccharide ligands to inhibit galectin-1 induced homotypic cellular aggregation in the A375 cell line [30]. Belitsky et al. designed self-assembled pseudopolyrotaxanes as a flexible and adaptable multivalent neoglycoconjugate for galectin-1 [31]. Using this multivalent supramolcular architecture, galectin-1 was observed to bind to flexible multivalent ligands with higher affinity than could be achieved using less dynamic ligand displays.

To further the mechanistic understanding of multivalent galectin-1 in biological processes such as cellular aggregation/tumor formation, we applied lactose functionalized poly(amidoamine) (PAMAM) dendrimers as a multivalent framework. We hypothesized that multivalent glycodendrimers would organize extracellular galectin-1 into aggregates that would influence the biological activity of galectin-1. To test this hypothesis, lactose functionalized dendrimers were used to nucleate the aggregation of galectin-1 into nanoparticles, and the sizes of the galectin-1/glycodendrimer nanoparticles were characterized using dynamic light scattering (DLS) and fluorescence microscopy (FM) when varying ratios of galectin-1 were added to the glycodendrimers. The galectin-1/glycodendrimer nanoparticle aggregates were then used to inhibit the galectin-1 induced aggregation of DU145 human prostate carcinoma cells. The studies reported here indicate that the pattern of galectin-1 that is presented to the cells influences their behavior, thus advancing the understanding of the mechanism of action of galectin-1 mediated cellular aggregation processes and indicating that multivalent interactions can be very effectively used to organize proteins into biologically active arrays.

## Results

### Nanoparticle formation

**Figure 2:**
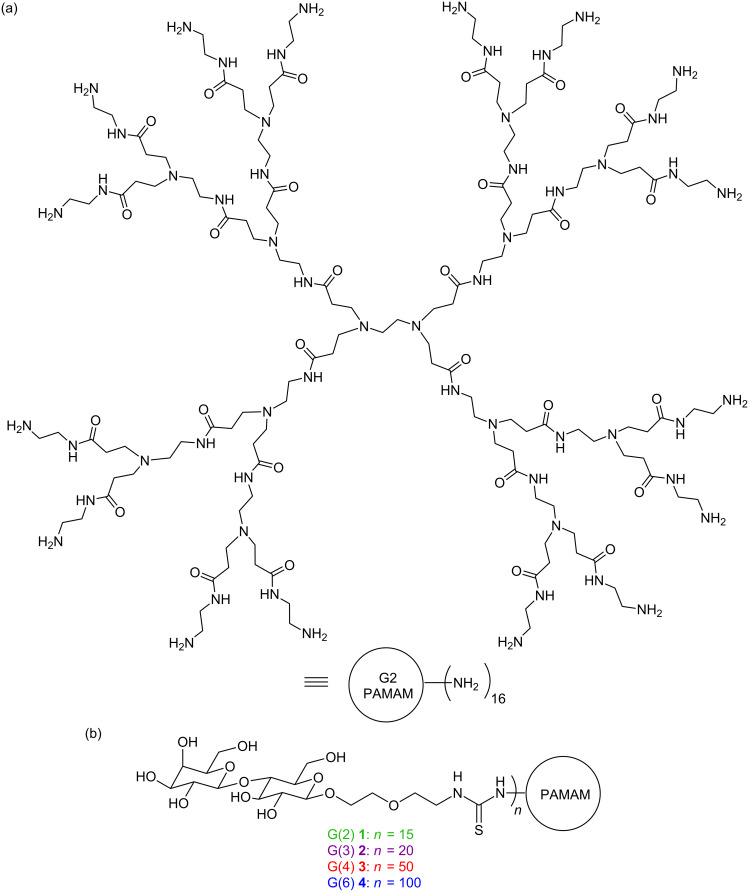
(a) Generation 2 PAMAM dendrimer. (b) Lactose-functionalized dendrimers **1**–**4**. Color-coding corresponds to colors used in the figures throughout this publication to indicate the different glycodendrimer generations.

Poly(amidoamine) (PAMAM) dendrimers were used as a multivalent framework to study multivalent protein–carbohydrate interactions. The PAMAM structure is shown in Figure 2a. Second, third, fourth, and sixth generation dendrimers were functionalized with lactoside endgroups using a bis-ethoxy linker for solubility to afford **1**–**4** (G(2), G(3), G(4), and G(6), respectively, Figure 2b) [32].

The sizes of the galectin-1/glycodendrimer nanoparticles that were formed using multivalent lactose-functionalized PAMAM dendrimers **1**–**4** were determined by fluorescence microscopy (FM) and dynamic light scattering (DLS). For fluorescence microscopy, galectin-1 was labeled with AlexaFluor-555, and aggregation was characterized when a large, medium, or slight excess of galectin-1 was used relative to the concentration of the dendrimer (220:1, 9:1, or 3:1 ratio of galectin-1 to dendrimer, respectively). Fluorescent microsphere standards (FluoSpheres Fluorescent Microspheres, Molecular Probes) and image analysis software (Pixcavator 6.0) were used for size quantifications.

**Figure 3:**
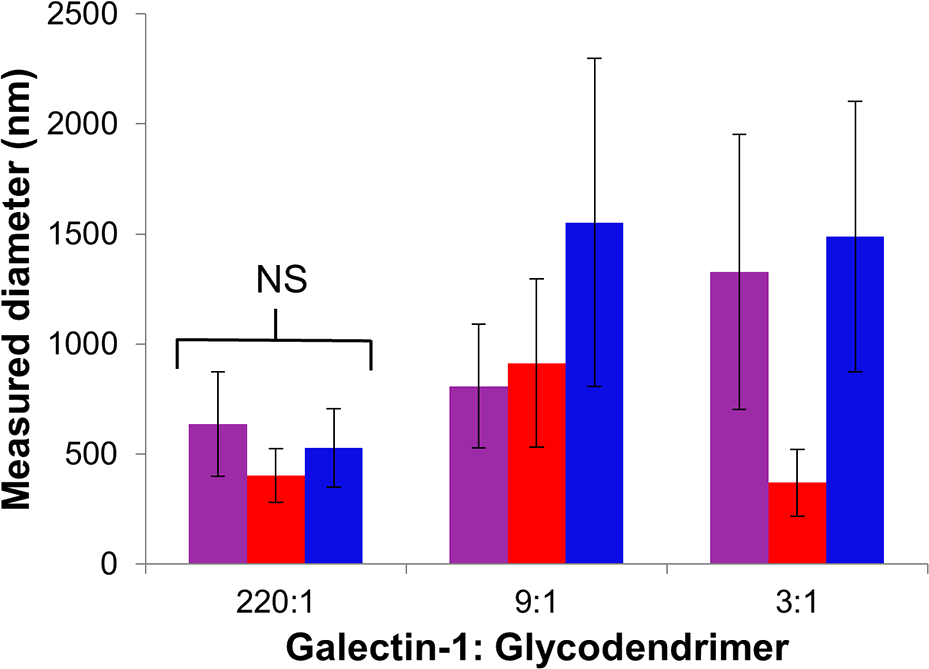
Average diameter (nm) of multivalent galectin-1 nanoparticles formed with multivalent glycodendrimers. For compounds **2** (purple), **3** (red), and **4** (blue), nanoparticle diameter (nm) was measured upon the addition of 0.18 µM glycodendrimer for 220:1, of 4.5 µM glycodendrimer for 9:1, and of 13 µM glycodendrimer for 3:1 to 40 µM galectin-1. NS represents non-significant difference in aggregate size measured for all generations determined by ANOVA.

**Figure 4:**
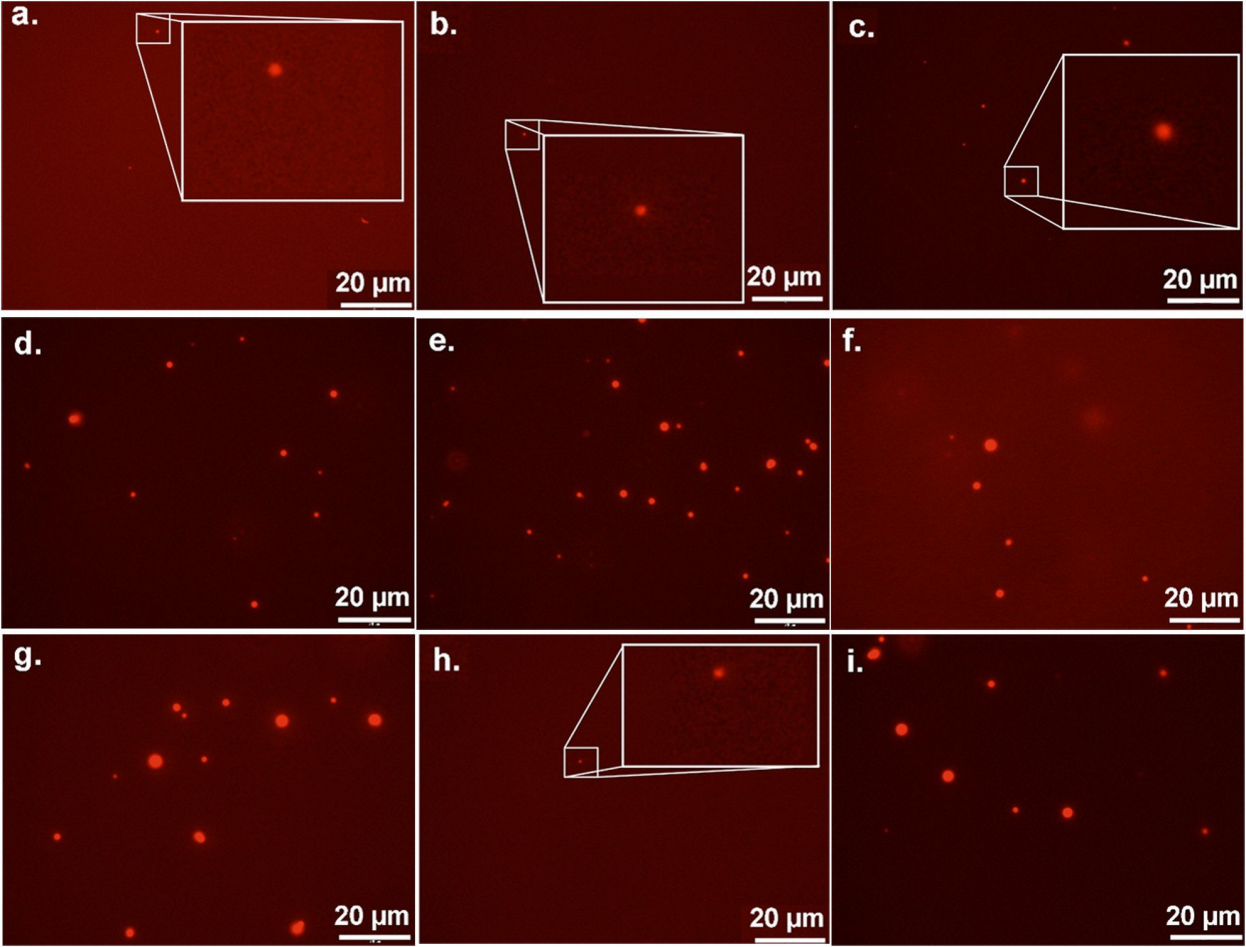
Representative fluorescent micrographs of glycodendrimer mediated galectin-1 nanoparticles. Nanoparticles formed with compounds (a) **2**, (b) **3**, and (c) **4** in a 220 molar excess of galectin-1 are shown in the top row and magnified by 4× for visualization. Nanoparticles formed with compounds (d) **2**, (e) **3**, and (f) **4** in a 9 molar excess of galectin-1 are shown in the middle row. In the bottom row, nanoparticles formed with compounds (g) **2**, (h) **3** (magnified by 4× for visualization), and (i) **4** in a 3 molar excess of galectin-1 are shown.

The results from the fluorescence microscopy studies using **2**, **3**, and **4** are summarized in Figure 3 (see Supporting Information File 1 for tabulated data), and representative micrographs are shown in Figure 4. (Aggregates formed using **1** were below the detection limits of the technique.) In the presence of a large excess of galectin-1 (220:1), all of the glycodendrimers **2**, **3**, and **4** organized galectin-1 into relatively small, similarly sized nanoparticles (Figure 4a–c). When a 9:1 or a 3:1 ratio of galectin-1 to glycodendrimer was used (Figure 4d–f and 4g–i), the aggregates that formed were generally larger and more polydisperse than when a 220-fold excess of galectin-1 was used. Only fourth generation dendrimer **3** forms comparable aggregates regardless of whether a slight excess of galectin-1 or a large excess of galectin-1 is added.

**Figure 5:**
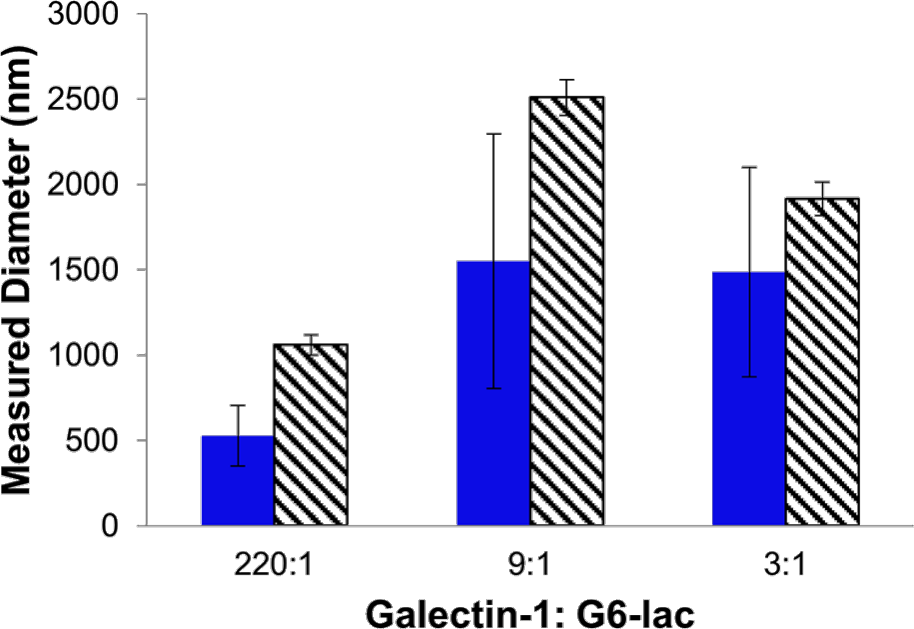
Comparison of average nanoparticle diameter (nm) formed with **4** measured by FM (blue) and DLS (diagonal stripes).

DLS was used as a complementary technique to characterize galectin-1 nanoparticles formed using **4**. These results, shown in Figure 5, also indicate the formation of small, homogeneous nanoparticles when a large excess of galectin-1 (220:1) was used. In agreement with the results obtained from the fluorescence microscopy studies, the nanoparticle sizes that were determined by DLS were larger when smaller-fold excesses of galectin-1 were used. Fluorescence microscopy proved to be a more robust technique for characterization of galectin-1 nanoparticles; galactin-1 nanoparticles formed using **2** and **3** exceeded the detection limits of DLS.

**Figure 6:**
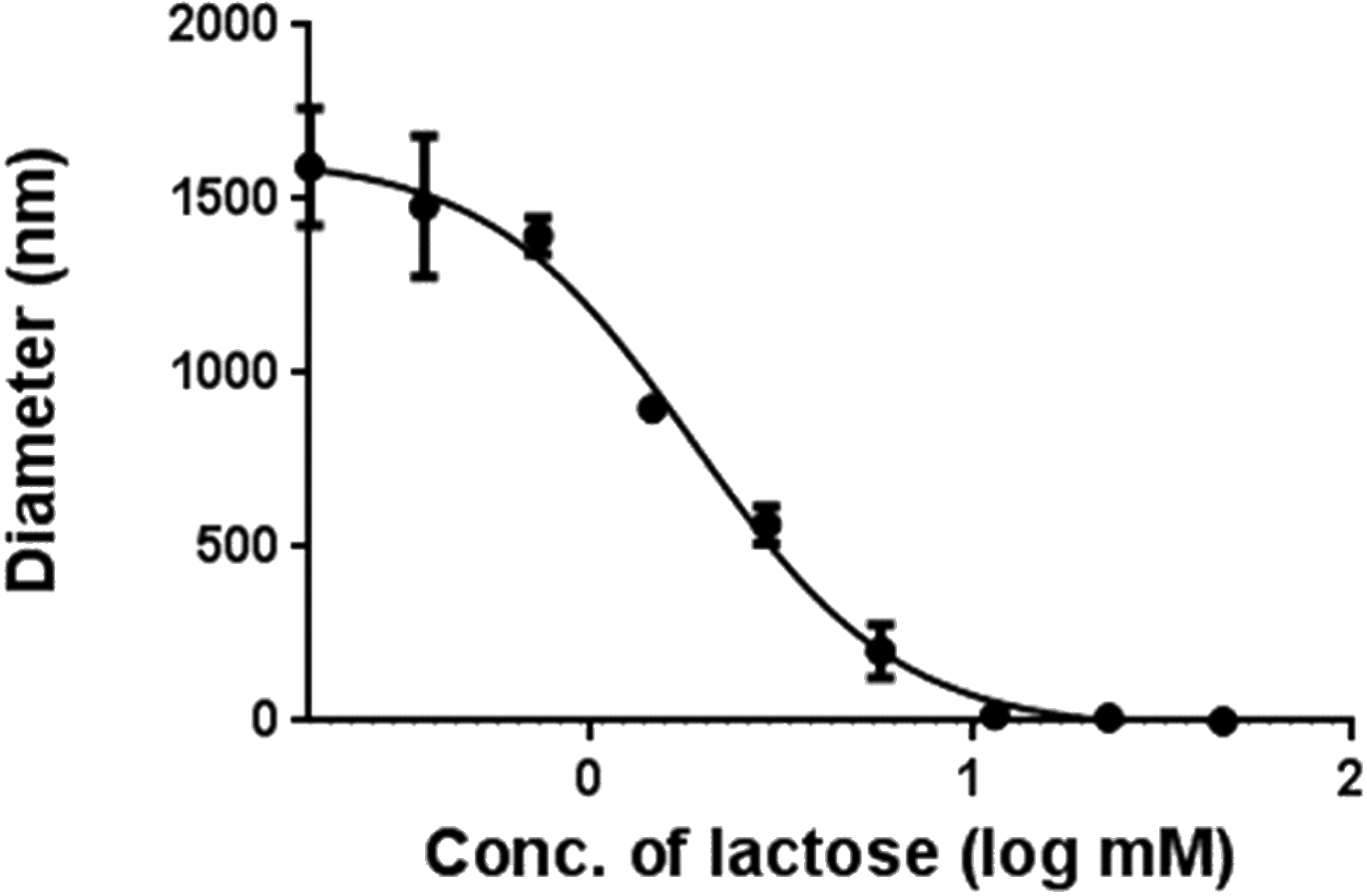
Lactose inhibition of galectin-1 nanoparticle formation with compound **4**.

Using DLS, the specificity of the interaction between galectin-1 and the lactosides on the multivalent glycodendrimers was assessed. Serially diluted solutions of monomeric lactose were co-incubated with galectin-1 and compound **4**. Complete inhibition of aggregation was achieved by monomeric lactose, with an IC_50_ of 1.9 mM, indicating that a specific interaction between the lactose endgroups on the dendrimers and the carbohydrate recognition site of galectin-1 occurs when nanoparticles are formed (Figure 6).

Control experiments were performed with different functional groups on the multivalent framework. No aggregates were detected upon the addition of a polyhydroxylated sixth generation dendrimer, indicating that binding requires more than merely an array of hydrogen bonds. Small nanoparticles (340 ± 20 nm) were obtained when mannose functionalized G(6)-PAMAMs were combined with galectin-1, and neither monomeric lactose nor monomeric mannose inhibited the formation of these aggregates. This indicates that nanoparticles formed using the mannose-functionalized dendrimer do not rely on interactions in the β-galactoside binding site on galectin-1 and that non-specific glycodendrimer/galectin-1 interactions are responsible for the formation of these small aggregates. Miller et al. observed galectin-1 binding to α-galactomannan derivatives, and NMR was used to determine that the interaction did not occur in the canonical CRD [33].

### Cell-based assay

After determining that lactose-functionalized dendrimers **1–4** reproducibly nucleate formation of galectin-1 aggregates that are quite homogeneous, we used these nanoparticles in cellular aggregation assays with galectin-1 and DU145 human prostate cancer cells. The DU145 cell line was chosen because it expresses a putative galectin-1 ligand – the Thomsen Friedenreich (TF) antigen on Mucin-1 [34–35].

**Figure 7:**
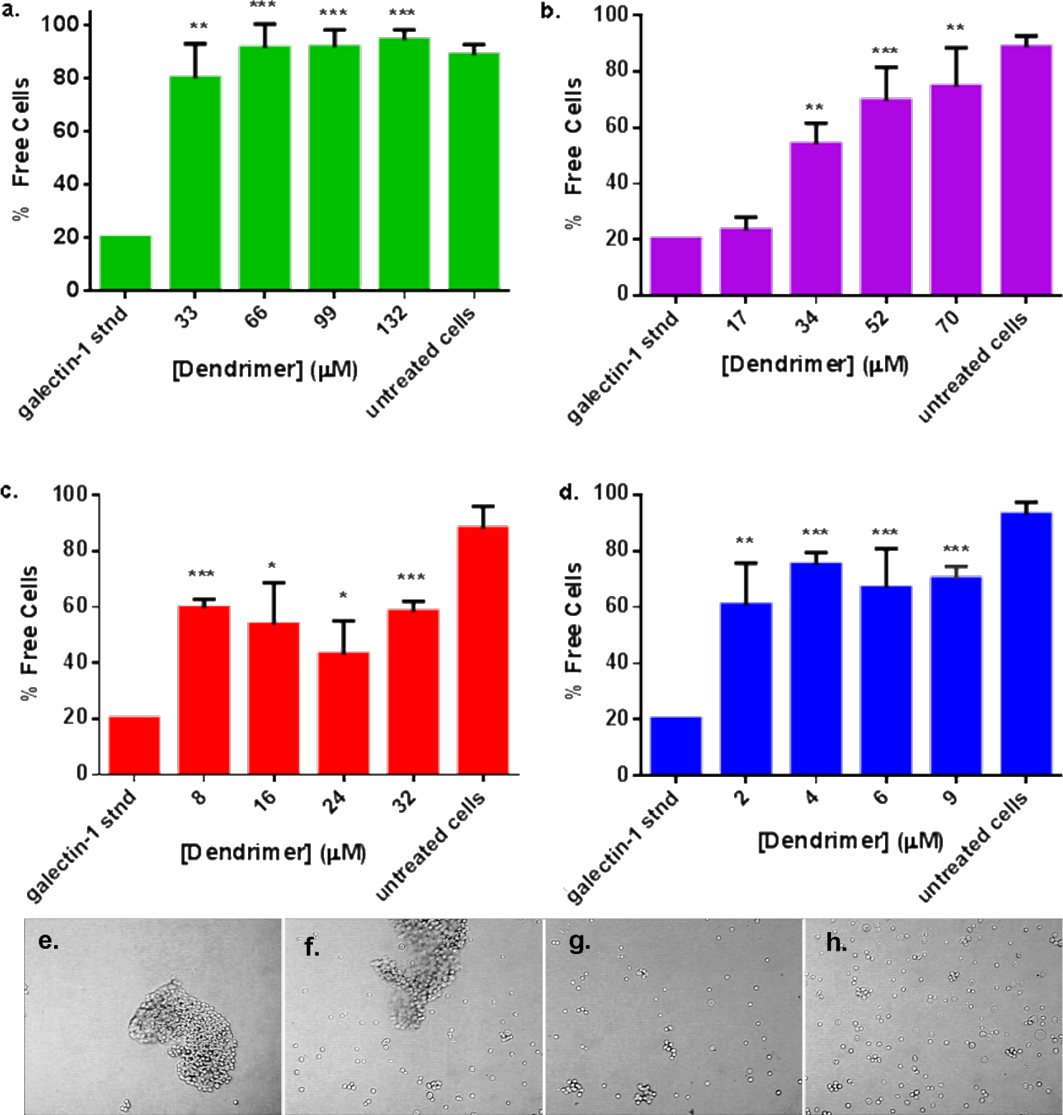
Cellular aggregation assays with DU145 human prostate carcinoma cells. Cancer cell aggregation assays were performed in the presence of 3.7 µM galectin-1 and increasing glycoderdrimer concentrations, with controls for galectin-1 treated cells and untreated cells. Glycodendrimer concentrations were normalized to present the same concentration of lactose residues. The results show inhibition of galectin-1 induced aggregation by (a) **1,** (b) **2**, (c) **3**, and (d) **4**. Data are shown as mean ± S.D. of measurements from at least three experiments. Statistical analysis was performed using an unpaired two-tailed student’s T-Test by comparing the % free cells to the galectin-1 standard and * indicates *p* value < 0.05, ** indicates *p* < 0.01, and *** indicates *p* < 0.001, representative images of cellular aggregation are provided for DU145 with 3.7 µM galectin-1 and: (e) 17 µM **2**; (f) 34 µM **2**; (g) 52 µM **2**; and (h) 70 µM **2**.

As shown in Figure 7, untreated DU145 cells were not aggregated (i.e., free cells); upon the addition of exogenous galectin-1, however, extensive aggregation was observed. When lactose functionalized dendrimers **1–4** were added to the DU145 cells with galectin-1, cellular aggregation was inhibited. The smallest glycodendrimer, second generation compound **1**, most effectively inhibited cellular aggregation. Even at the lowest concentration of **1** shown in Figure 7, complete inhibition of cellular aggregation was observed (Figure 7a). Incomplete inhibition of aggregation was observed for compounds **3** and **4**. For fourth generation lactose functionalized dendrimer **3**, the percentage of free cells plateaued at 50% (Figure 7c). With sixth generation lactose functionalized dendrimer, **4**, only 30% of the cells remained clustered (Figure 7d). Although glycodendrimer concentrations were normalized so that the same concentration of lactoside residues were present at each stage in the assay irrespective of the scaffold generation number, dose-responsive inhibition of galectin-1 mediated cancer cell adhesion was only observed with lactose functionalized G(3)-dendrimer **2** at these concentrations (Figure 7b, and representative images 7e–h, the dose-responsive curve for lower concentrations of **1** is provided in Supporting Information File 1). Nearly complete inhibition of cellular aggregation was observed with compound **2** at the highest concentration of **2**. The inhibition observed with compounds **1** and **2** indicates that the smaller glycodendrimers are the most effective inhibitors of galectin-1 induced cellular aggregation.

A control experiment was performed to measure the ability of monomeric lactose to inhibit aggregation of DU145 cells in the presence of 3.7 µM exogenous galectin-1. The concentration of monomeric lactose required to inhibit cellular aggregation is 6 mM. On a per lactose basis, this concentration is 15-fold higher than the 66 µM concentration of **1** that was required for complete inhibition of cellular aggregation. Additionally, mannose-functionalized G(6)-PAMAM dendrimers did not inhibit cellular aggregation.

## Discussion

**Figure 8:**
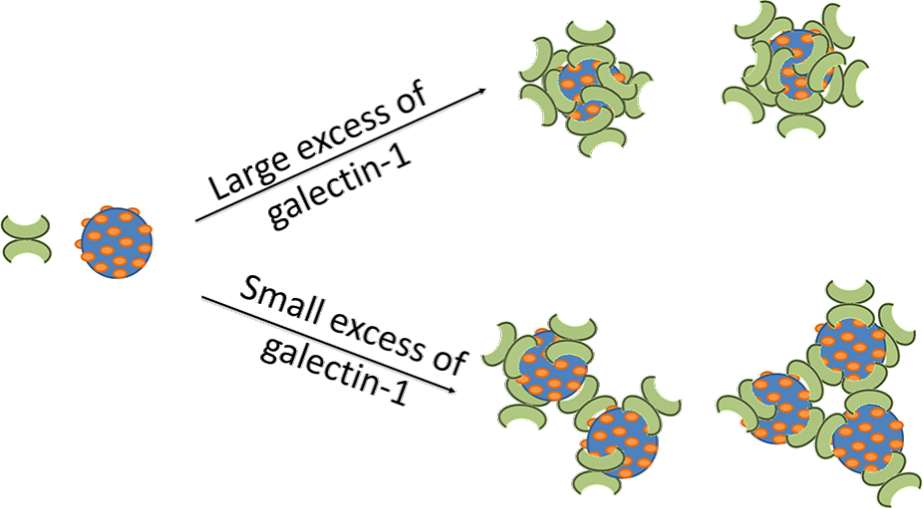
Schematic representation of galectin-1/glycodendrimer aggregates at varying stoichiometries.

The results of the fluorescence microscopy and DLS studies described above reveal that multivalent glycodendrimers organize galectin-1 into nanoparticles. In the presence of a large excess of galectin-1, multivalent glycodendrimers **2–4** organize galectin-1 into relatively small and remarkably homologous nanoparticles (Figure 3 and Figure 4a–c). This is likely a result of the multivalent framework being saturated with galectin-1, providing few uncomplexed nucleation sites for incorporation into larger nanoparticles (Figure 8). Therefore, an increase in the concentration of the multivalent framework should correlate to an increase in aggregate size, as was observed for 9:1 and 3:1 ratios of galectin-1 to glycodendrimer (Figure 3 and Figure 8). The exception to this is that small homogeneous nanoparticles were observed for compound **3** when a slight excess of galectin-1 was used (3:1). In this lactoside-rich environment, the presence of a large excess of lactoside residues apparently enabled increased nucleation at the expense of aggregation, but it isn’t clear why **3** is different from the other dendrimers in this regard. Overall, the results described here agree with mathematical modeling studies that identified scaffold concentration as a key determinant in maximizing scaffold-mediated nucleation [36].

The size of the nanoparticles formed in the presence of a large excess of galectin-1 is fundamentally remarkable. In the presence of enough galectin-1 to saturate the multivalent framework, aggregates approximately 400 nm in diameter were measured (Figure 3). The distance between the galectin-1 CRDs is approximately 5 nm [21]. The diameter of the G(3), G(4), and G(6)-PAMAM dendrimers used to synthesize compounds **2**, **3**, and **4**, respectively, range from approximately 4 nm to 7 nm [37]. Therefore, multiple galectin-1 and glycodendrimer particles must interact to form the 400 nm aggregates, and this is favorable even when the scaffold is ostensibly saturated with galectin-1.

The DU145 human prostate carcinoma cell line was chosen to demonstrate that multivalent interactions initiated by a synthetic multivalent system can be used for effectively controlling cellular processes. DU145 cells express elevated levels of both galectin-1 [38] and its putative receptor Mucin-1 [34], which suggests that galectin-1 mediated β-galactoside binding is critical to cellular aggregation/tumor formation in this cell line. In the presence of exogenous galectin-1, extensive cellular aggregation was observed. Inducement of aggregation by exogenous galectin-1 comports with literature reporting pro-adhesive activity with galectin-1 [38–41]. There are two likely mechanism for galectin-1 mediation of cellular aggregation: (i) cross-linking of glycoconjugates (TF antigen Mucin-1) on adjacent cells which directly facilitates aggregation; and (ii) clustering of receptors (TF antigen Mucin-1) which exposes adhesion molecules that interact with adhesion molecules on neighboring cells to cause aggregation.

All four generations of the glycodendrimers inhibited galectin-1 mediated cellular aggregation of the DU145 cells, which indicates that glycodendrimers mediate inhibition of cellular aggregation by competitively binding galectin-1, thereby altering its presentation to cells and preventing cellular cross-linking. Lactose functionalized G(2)-PAMAM **1** was the most potent inhibitor of galectin-1 induced cellular aggregation, exhibiting complete inhibition of cancer cell adhesion at low dosage (Figure 4). Galectin-1/**1** nanoparticles were not detected by DLS or fluorescence microscopy. Because galectin-1 is known to bind these glycodendrimers, it is likely that aggregates formed but were below the detection limit of the fluorescence microscopy technique (which is about 200 nm). The formation of aggregates smaller than 200 nm in diameter, and thus not detectable by fluorescence microscopy, lends further credence to the argument that small galectin-1/glycodendrimer aggregates effectively alter the presentation of galectin-1 to cells, thereby altering the cells’ recognition events.

Inhibition by monomeric lactose well illustrates the multivalency avidity enhancement. Monomeric lactose inhibited cell adhesion at a concentration of 6 mM, while inhibition of cellular adhesion by **1** occurred at a lactose concentration of 0.4 mM. This is a 15-fold increase in the concentration of lactose required to disrupt galectin-1 mediated cancer cell adhesion compared to the multivalent counterpart. The pronounced inhibition suggests that multivalent glycodendrimers **1–4** have a strong influence on the native cellular adhesion mechanism.

## Conclusion

The concept that multivalency can be used to effectively control cellular activities was investigated using lactose functionalized dendrimers. First, the ability of the multivalent framework to organize galectin-1 was assessed with dynamic light scattering and fluorescence microscopy. These studies indicate that multivalent glycodendrimers nucleate the aggregation of galectin-1 into nanoparticles, which were remarkably homogenous when formed in the presence of a large excess galectin-1. Next, glycodendrimers were added to cancer cells to modulate galectin-1 mediated cellular aggregation. The glycodendrimers inhibited cellular aggregation by providing competitive binding sites for the galectin-1 and diverting the galectin-1 from its native role in cellular cross-linking, which leads to cellular aggregation/tumor formation. These studies reveal that mutivalency can be exploited not only to modulate biological activities but also as a platform to advance the understanding of biologically relevant protein/carbohydrate interactions through the ability to organize proteins into biologically active arrays.

## Experimental

### General information

Galectin-1 was provided by Dr. Linda Baum and Mabel Pang of UCLA. General reagents were purchased from Acros and Sigma-Aldrich Chemical Companies. PAMAM dendrimers were purchased from Dendritech. The lactose-functionalized dendrimers used (**1**–**4**) were synthesized and characterized according to the reported procedure [32].

### Fluorescence microscopy

Reagents for fluorescence microscopy were purchased from Molecular Probes. To measure galectin-1 nanoparticles formed using glycodendrimers with fluorescence microscopy, both species were fluorescently labeled. Galectin-1 was labeled with Alexa Fluor A555 NHS Ester (succinimidyl ester) (Molecular Probes) [42]. Fluorescent images were captured on an Olympus BX-61 motorized microscope with MicroSuite software with a 100× oil immersion objective at an exposure time of 2 ms. Size quantification was achieved using fluorescent microsphere standards (200 nm, 1000 nm, and 10000 nm reported diameter) (FluoSpheres Fluorescent Microspheres, Molecular Probes) and image analysis software (Pixcavator 6.0). At a constant concentration of galectin-1 (40 µM), aggregate size was measured at ratios of galectin-1 to glycodendrimer of 220:1, 9:1, and 3:1.

### Dynamic light scattering

Dynamic light scattering was performed using a 90 Plus Particle Size Analyzer (Brookhaven Instruments Corp.) to measure galectin-1/glycodendrimer aggregates at same concentrations and ratios that were used in the fluorescence microscopy assays. Monomeric lactose was co-incubated with galectin-1 and compound **4** for inhibition assays. For controls, mannose-functionalized G(6)-PAMAM dendrimer [43] and a polyhydroxylated G(6)-PAMAM dendrimer (Dendritech) were used. Inhibition experiments using mannose functionalized G(6) were performed using monomeric mannoside and monomeric lactoside, respectively.

### Cell-based assay

Human prostate carcinoma cells (DU145, ATCC HTB-81) were purchased from ATCC, and maintained in Dulbecco’s modified Eagle’s medium (DMEM, Gibco) supplemented with 10% fetal bovine serum (FBS, Gibco). 2 mg/mL stock solutions of glycodendrimers were prepared in PBS buffer. Increasing glycodendrimer concentrations were added to a constant concentration of galectin-1 (3.7 µM) and cancer cells (≈240,000/eppendorf). Glycodendrimer concentrations were calculated to present approximately equal concentrations of lactosides residues at the same stage in the assays irrespective of PAMAM generation. Control assays for untreated cells (untreated standard) and the galectin-1 treated cells (galectin-1 standard) were performed. Control assays with the glycodendrimers and without galectin-1 were previously performed [44]. Assays were incubated at 37 °C and gently rotated for 1 hour. Images were captured on a Jenco microscope with 10× objective, and quantification was achieved using image analysis software (Pixcavator 6.0). Particles of fewer than five cells were defined as free cells and particles greater than five cells were defined as aggregated. Statistical analyses were performed using unpaired two-tailed student’s T-Test by comparison to the galectin-1 standard. Statistically significant data is represented as * if *p* < 0.05, ** if *p* < 0.01, and *** if *p* < 0.001. The interaction between the galectin-1 and the DU145 cells generated large aggregates that exceeded the detection limit of the technique. Visual inspection of galectin-1 treated cells confirmed nearly complete aggregation of all cells; therefore, the percentage of free cells for the galectin-1 treated DU145 cells without glycodendrimer (galectin-1 stnd) was conservatively set at 20%.

## Supporting Information

File 1 Experimental procedures, fluorescent micrographs of fluorescent standards and calibration curve, and statistical analysis of fluorescent microscopy results.
