# Supplementary material for: Glycodendrimers: tools to explore multivalent galectin-1 interactions
Source: Beilstein J Org Chem. 2015 May 12;11:739–47. doi: 10.3762/bjoc.11.84 (PMC4464428; doi:10.3762/bjoc.11.84)
Supplement: File 1 — Experimental procedures, fluorescent micrographs of fluorescent standards and calibration curve, and statistical analysis of fluorescent microscopy results. [file Beilstein_J_Org_Chem-11-739-s001.pdf]

Supporting Information

for

Glycodendrimers: tools to explore multivalent galectin-1 interactions

Jonathan M. Cousin and Mary J. Cloninger*

Address: Department of Chemistry and Biochemistry, Montana State University, Bozeman, MT 59717, USA

Email: Mary J. Cloninger - mcloninger@chemistry.montana.edu

* Corresponding author

Experimental procedures, fluorescent micrographs of fluorescent standards and calibration curve, and statistical analysis of fluorescent microscopy results

Table of Contents

	Page
General experimental protocol	S2
Fluorescence microscopy protocol	S2–S6
Fluorescence microscopy statistical analysis	S3–S4
Fluorescence microscopy calibration curve and micrographs for standard fluorescent microsphere	S4–S5
Dynamic light scattering protocol	S7–S10
Cell-based assay protocol	S10–S11
Representative cell images	S12–S15
Saturation curve for **1**	S15
Control experiments for cell based assays	S16
Solution preparation for cell based assays	S17
References for the supporting information	S17

General methods

Galectin-1 was provided by Dr. Linda Baum and Mabel Pang of UCLA in stock aliquots of 6.4 and 7.2 mg/mL and stored at −78 °C. General reagents were purchased from Life Technologies, Inc. PAMAM dendrimers were purchased from Dendritech.

Nanoparticle formation

Fluorescence microscopy

Fluorescent chromophores and fluorescent microsphere standards (FluoSpheres®) were purchased from Molecular Probes™. Fluorescent images were captured on an Olympus BX-61 motorized microscope with MicroSuite™ software with a 100× oil immersion objective. Exposure time was optimized at 2 ms. Gimp 2.8 image manipulation software was used to combine images for subsequent particle perimeter (pixel) analysis. Pixcavator 6.0 Image Analysis software (Intelligent Perception) was used to measure particle perimeter in terms of pixel count. Particles below a threshold of 80 “round” as calculated by Pixcavator were excluded from analysis.

To a final concentration of galectin-1 of 40 µM, varying amounts of glycodendrimer (0.18, 4.5, and 13 µM; final concentration) were added to achieve the following ratios of galectin-1 to glycodendrimer: (a) 220:1; (b) 9:1; and (c) 3:1. PBS was added to bring the total volume of the solution to 100 µL. Assays were incubated for 1 hour at room temperature (19 °C). Each sample was scanned in triplicate, at intervals of approximately five minutes and vortexed between scans. The average measured

diameter and polydispersity of the fluorescently labeled galectin-1/glycodendrimer aggregates is provided in Table S1.

Table S1: FM measured polydispersity and diameter (nm) of galectin-1/glycodendrimer aggregates.

Compound	Galectin-1 (μM)	Dendrimer (μM)	Galectin-1: Dendrimer	Average Dia. (nm)	Polydispersity
1	40	0.18	220:1	NDA*	
		4.5	9:1	NDA	
		13	3:1	NDA	
2	40	0.18	220:1	640 ± 340	1.2
		4.5	9:1	810 ± 280	1.1
		13	3:1	1300 ± 630	1.2
3	40	0.18	220:1	400 ± 140	1.1
		4.5	9:1	910 ± 380	1.2
		13	3:1	370 ± 160	1.2
4	40	0.18	220:1	530 ± 180	1.1
		4.5	9:1	1600 ± 750	1.2
		13	3:1	1500 ± 620	1.2

*NDA: No detectable aggregates

Fluorescence microscopy data was analyzed for statistically significant differences in aggregate size at the varying ratios (Figure S1). For each compound, statistically significant changes in aggregate size were determined by comparing the size of aggregates formed at 220:1 to 9:1 and those formed at 9:1 to 3:1 (Table S3). In addition, for each compound, aggregates formed in the presence of a slight excess of galectin-1 (3:1) were analyzed for statistical significance with respect to aggregates formed in the presence of a large excess (220:1). The size of galectin-1 aggregates formed with compounds **2**, **3**, and **4**, respectively, were compared at 220:1.

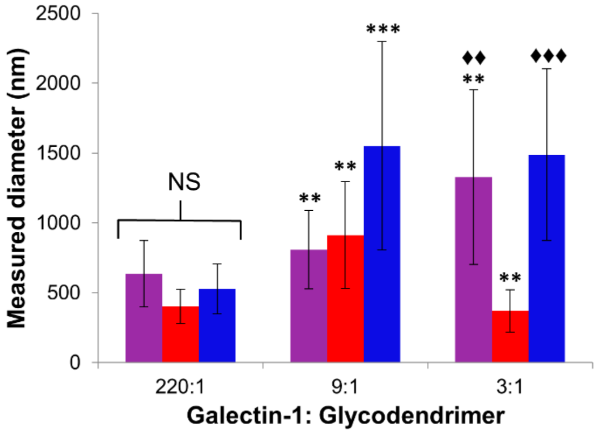

Figure S1: Average diameter (nm) of multivalent galectin-1 nanoparticles formed with multivalent glycodendrimers. Nanoparticle diameter (nm) was measured in the presence of galectin-1 at 220 molar excess, a 9 molar excess, and a 3 molar excess with compounds **2** (purple), **3** (red), and **4** (blue), respectively. Statistical analyses were performed using a paired two-tailed student's T-Test. Symbols (* and ♦) are used to indicate a statistically significant change in the size aggregates as the ratio of galectin-1 changes relative to a compound. The symbols *, **, and *** indicate a statistical significance of $p < 0.05$, $p < 0.01$, and $p < 0.001$, respectively, by comparing aggregates formed at 9:1 with respect to aggregates formed at 220:1, and by comparing aggregates formed at 3:1 with respect to those formed at 9:1. The symbols ♦, ♦♦, and ♦♦♦ indicate a statistical significance of $p < 0.05$, $p < 0.01$, and $p < 0.001$, respectively, by comparing aggregates formed at 3:1 with respect to those formed at 220:1. NS represents non-significant difference in aggregate size measured at 220:1 for all generations determined by ANOVA.

Pixel (count) to size (nm) calibration curve

Fluorescent microsphere standards (FluoSpheres®, Molecular Probes™) were used to establish a calibration curve to convert the measured particle perimeter (pixels) to diameter (nm) at the optimized exposure time (Figure S2). Representative images of the fluorescent microsphere standards are shown Figure S3a–c. Table S2 shows the effective diameter of the fluorescent microspheres as measured with DLS.

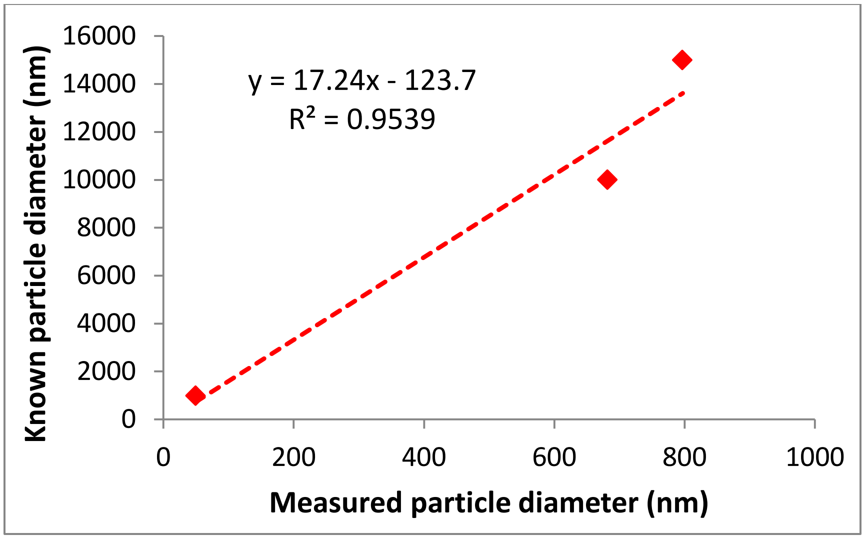

Figure S2: Calibration curve correlating measured pixels to reported particle diameter (nm).

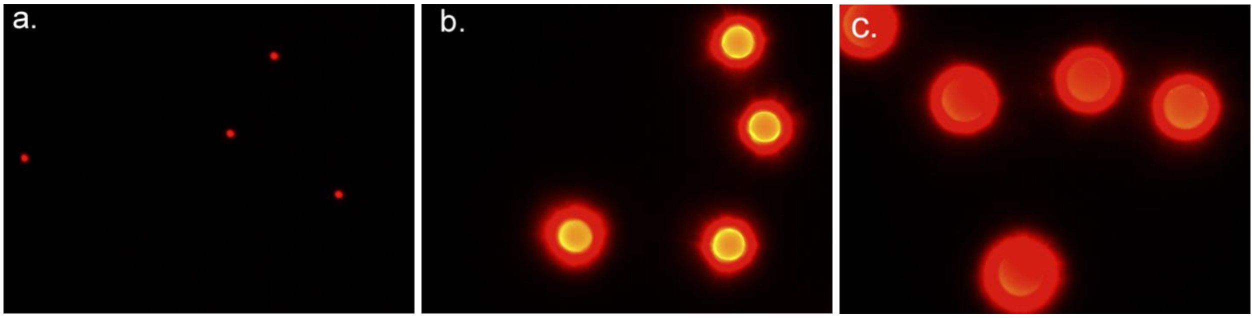

Figure S3: Representative fluorescent micrographs for fluorescent microsphere standards. (a) 200 nm fluorescent microsphere standard. (b) 1000 nm fluorescent microsphere standard. (c) 10000 nm fluorescent microsphere standard.

Table S2: DLS measured diameter (nm) for standard fluorescent microspheres.

Reported Diameter of Standard (nm)	Measured Effective Diameter (DLS, nm)	Polydispersity
200	230 ± 11	0.051 ± 0.047
1000	1100 ± 20.0	0.005 ± 0.0
10000	9800 ± 260[a]	2.9 ± 0.57[a]

[a]Results from NNLS analysis.

Assays were run in triplicate and incubated for 1 hour at room temperature. From each sample, three 10 μL aliquots were spotted on a microscope slide. Each spot was then viewed with a Fluorescent Microscope (Olympus BX-61 motorized microscope with MicroSuite™ software with a 100× oil immersion objective). At least three pictures were taken for each spot. Images were acquired immediately upon observation of a fluorescent particle.

Alexa Fluor® 555 labeled galectin-1

For a 2 mg/mL stock solution, Alexa Fluor® 555 NHS powder (1 mg) was dissolved in 500 mL millipore H_2O. Galectin-1 was prepared in PBS in stock solutions of 3.6 mg/mL. To the galectin-1 solution, Alexa Fluor® 555 (6 equiv) was added and the reagents were allowed to react at room temperature (19 °C) for 1 h in PBS buffer. The reaction mixture was purified by dialysis using 1 kD MWCO dialysis tubing (Spectrum Laboratories, Inc., 6 Spectra/Por® Dialysis Membrane) in PBS buffer. Characterization of galectin-1 labeling was achieved in terms of a change in absorbance using a UV–vis Spectrometer (Molecular Devices, SpectraMax Plus, Softmax® Pro 5). Absorbance was measured at 555 nm with a molar extinction coefficient of 150,000 $M^{-1}cm^{-1}$.

Dynamic light scattering (DLS)

Galectin-1/glycodendrimer aggregate diameter measurements using DLS were acquired on a Brookhaven 90PLUS Particle Size Analyzer with a 15 mW solid state, 633 nm laser and APD detector. Scattered light was detected at 90° incidence and the signal intensity was set to the maximum kilocounts per second. Temperature control was stabilized at 25 °C. Samples were scanned over a 5 minute time range. Brookhaven software was used to analyze autocorrelation curves using the method of cumulants (quadratic fit) unless stated otherwise. The method of cumulants analysis provided results similar to NNLS and CONTIN algorithms for samples that were relatively monodisperse with relatively high signals.

Prior to mixing the reagents, all solutions were filtered using a 0.22 µm millipore filter (Millex® - GV 0.22 µm Syringe-Driven Filter Unit) to eliminate dust particle interference. Assays were run at total volume of 200 µL in a UV-cuvette (Eppendorf UVette®, 50–2000 µL, 220–1600 nm, center height 8.5 nm). Galectin-1 stock solutions were prepared in PBS. To a final concentration of galectin-1 of 40 µM, varying amounts of glycodendrimer (0.18, 4.5, and 13 µM; final concentration) were added to achieve the following ratios of galectin-1 to glycodendrimer: (a) 220:1; (b) 9:1; and (c) 3:1. PBS was added to the UV-cuvette to bring the total volume of the solution to 200 µL.

Assays were incubated for 1 h at room temperature (19 °C). Each sample was scanned in triplicate, at intervals of approximately five minutes, and vortexed between scans. The measured effective diameter (nm), polydispersity, and signal (kcps, kilocounts per second) for each scan were averaged for 3–6 samples and reported below in Table S3.

Table S3: DLS signal and measured galectin-1/glycodendrimer effective diameter (nm).

Compound	Galectin-1 (µM)	Dendrimer (µM)	Galectin-1: Dendrimer	Effective Dia. (nm)	Polydispersity	Signal (kcps)
1	40	0.18	220:1	NDA		
		4.5	9:1	NDA		
		13	3:1	NDA		
2	40	0.18	220:1	NDA		
		4.5	9:1	NDA		
		13	3:1	NDA		
3	40	0.18	220:1	NDA		
		4.5	9:1	NDA		
		13	3:1	NDA		
4	40	0.18	220:1	1090 ± 60[a]	0.44 ± 0.46	56 ± 35
		4.5	9:1	2510 ± 110	0.16 ± 0.07	194 ± 65
		13	3:1	1860 ± 100	0.29 ± 0.11	40 ± 6

[a]Results from Contin analysis;
NDA: No detectable aggregates.

Lactose inhibition

A lactose inhibition assay was performed in which monomeric lactoside was co-incubated with galectin-1 and compound **4**. Assay solutions were prepared at a final concentration of 40.0 µM galectin-1 and 0.35 µM **4** in a final volume of 200 µL. Separate samples were prepared for each concentration of monomeric lactoside tested. Three trials were prepared for each concentration, and triplicate measurements were performed for each sample at approximately five minute intervals. Lactose inhibition measurements are reported in Table S4.

Table S4: DLS signal and aggregate diameter (nm) measured for monomeric lactose inhibition of galectin-1 (40 µM) /G6-Lac **4** (0.39 µM) aggregate formation.

[Lactose] (mM)	Log [Lactose (mM)]	Effective Diam. (nm)	Polydispersity	Signal (kcps)
0.18	- 0.74	1600 ± 240	0.170 ± 0.089	40 ± 30
0.36	- 0.44	1480 ± 290	0.135 ± 0.085	40 ± 20
0.72	- 0.14	1400 ± 240	0.130 ± 0.083	60 ± 45
1.4	0.16	910 ± 40	0.168 ± 0.052	20 ± 5
2.9	0.46	600 ± 70	0.241 ± 0.094	5 ± 2
5.8	0.76	200 ± 90	0.562 ± 0.302	5 ± 5
12	1.1	30 ± 27	0.550 ± 0.204	3 ± 2
24	1.4	20 ± 10	1.0 ± 1.0	1.5 ± 0.5
48	1.7	NDA		

NDA: No detectable aggregates.

Controls

To galectin-1 (40 µM final concentration), mannose functionalized G(6) dendrimer (3.1 µM final concentration) was added. Aggregates of 340 ± 21 nm diameter were measured. Separate monomeric mannose and monomeric lactose inhibition experiments were performed to inhibit the galectin-1 aggregate formed with the mannose functionalized dendrimer. Additionally, to a final concentration of galectin-1 of 40 µM, polyhydroxylated G(6)-PAMAM was added, and no aggregate was detected. (Particles of a diameter 6.8 ± 0.38 nm as measured by NNLS analysis agree with the reported value for polyhydroxylated G(6)-PAMAM dendrimer)[1].

Solution preparation

Galectin-1 was prepared in filtered PBS. Lactose functionalized dendrimers (compounds **1, 2, 3,** and **4**), mannose functionalized G(6)-PAMAM dendrimer, and a polyhydroxylated dendrimer G(6)-PAMAM were prepared by dissolving a lyophilized

sample in filtered Millipore water to a final concentration of 10 µM and 100 µM. Monomeric lactoside was dissolved in filtered PBS buffer to afford a 100 µM solution.

Cell-based assays

The human prostate carcinoma cell line DU145 was purchased from ATCC® (HTB-81™). Cellular aggregation assays were observed on a Jenco™ USA microscope with 10× objective and a Canon Powershot A630 camera at 4× magnification. All reagents for cell-based media were purchased from Gibco® by Life Technologies™, except $NaHCO_3$, which was purchased from Fisher Scientific.

Adhered cells were removed with 0.5 mM EDTA and collected in a 15 mL Falcon tube. After sufficient centrifugation to form a cell pellet, the EDTA solution was removed and the pellet was re-suspended in 1 mL serum free media (SFM). Cells were counted using a hemocytometer, following a 10× dilution in SFM of a 10 µL aliquot of the cell suspension and a subsequent 2× dilution in Trypan Blue (TPB) of 10 µL aliquot of the previously diluted sample. Centrifugation and dilution of the cells in SFM were performed to achieve a concentration of 16×10^6 cells/mL. 15 µL of the cell suspensions were added to each Eppendorf for a final concentration approximately 240,000 cells/70 µL. For all assays, other than the untreated control, a final galectin-1 concentration of 3.7 µM was used. The volume of glycodendrimer (compounds **1–4**) added was doubled at each stage in the assay, and SFM was added to bring the final volume to 70 µL. Table S5 below contains the volume of the reagents added for the untreated control (Tube 1), galectin-1 with increasing glycodendrimer concentrations (Tubes 2–5), and galectin-1 control (Tube 6).

Table S5: Cell-based assay. Tube 1: untreated DU145 control; Tubes 2–5: DU145 cells with galectin-1 and increasing glycodendrimer; and Tube 6: galectin-1 treated control.

Tube	1	2	3	4	5	6
Dendrimer (µL)	0	10	20	30	40	0
Galectin-1 (µL)	0	15	15	15	15	15
Cells (µL)	15	15	15	15	15	15
SFM (µL)	55	30	20	10	0	40

Assays were run in triplicate. From each Eppendorf tube, three 10 µL aliquots were spotted on a microscope slide. At 4× magnification, 4 images were taken from each spot, for a total of 36 images. 12 images were stitched together and converted to black cells and white background for quantification by Pixcavator 5.0. Representative black and white cell images are provided in Figures S4–S7.

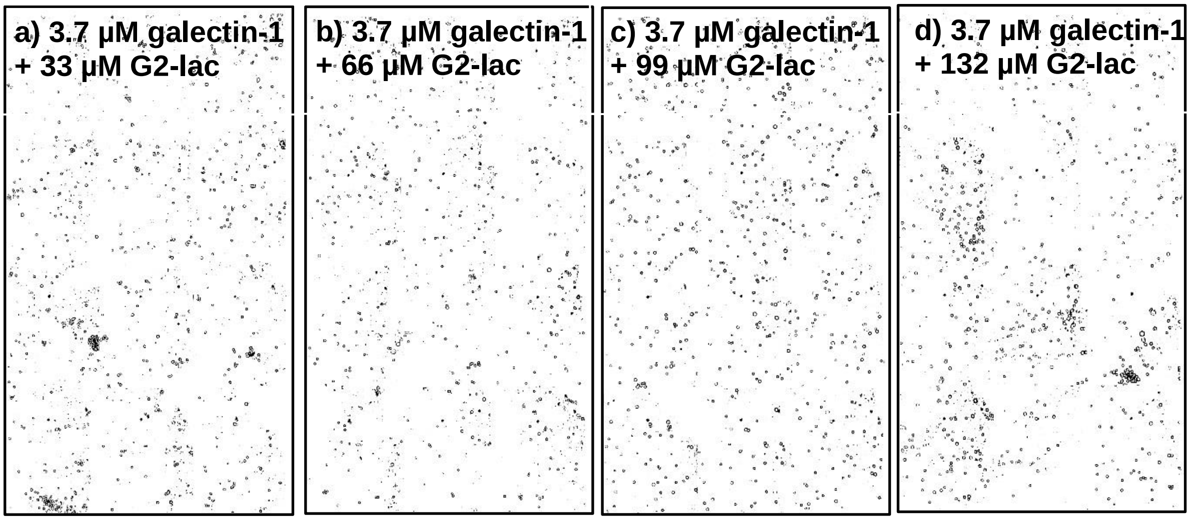

Figure S4: Inhibition of galectin-1 mediated aggregation of DU145 cells by (a) 33 μM **1**, (b) 66 μM **1**, (c) 99 μM **1**, and (d) 132 μM **1**.

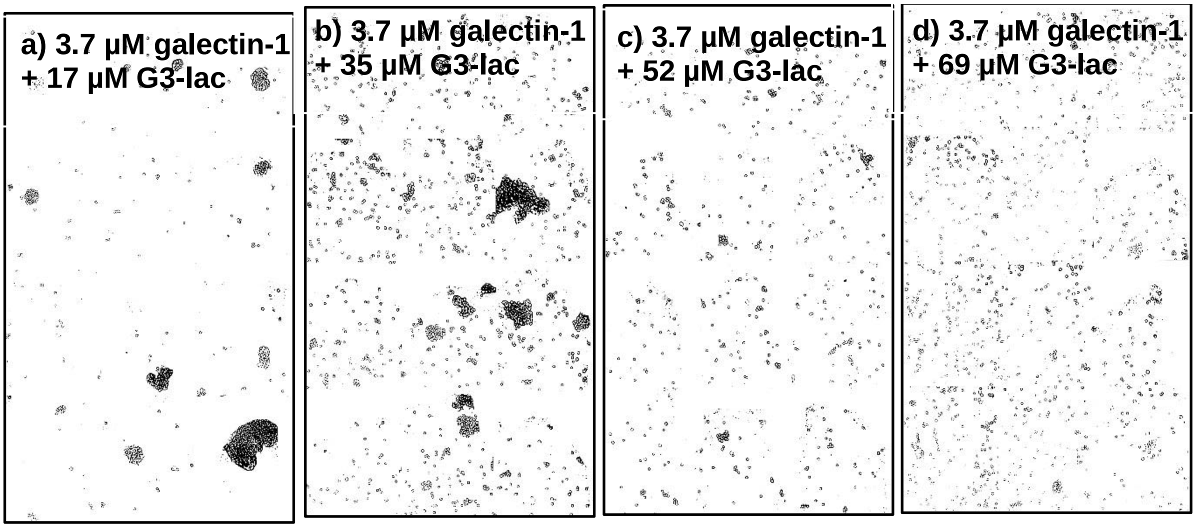

Figure S5: Inhibition of galectin-1 mediated aggregation of DU145 cells by (a) 17 μM **2**, (b) 35 μM **2**, (c) 52 μM **2**, and (d) 69 μM **2**.

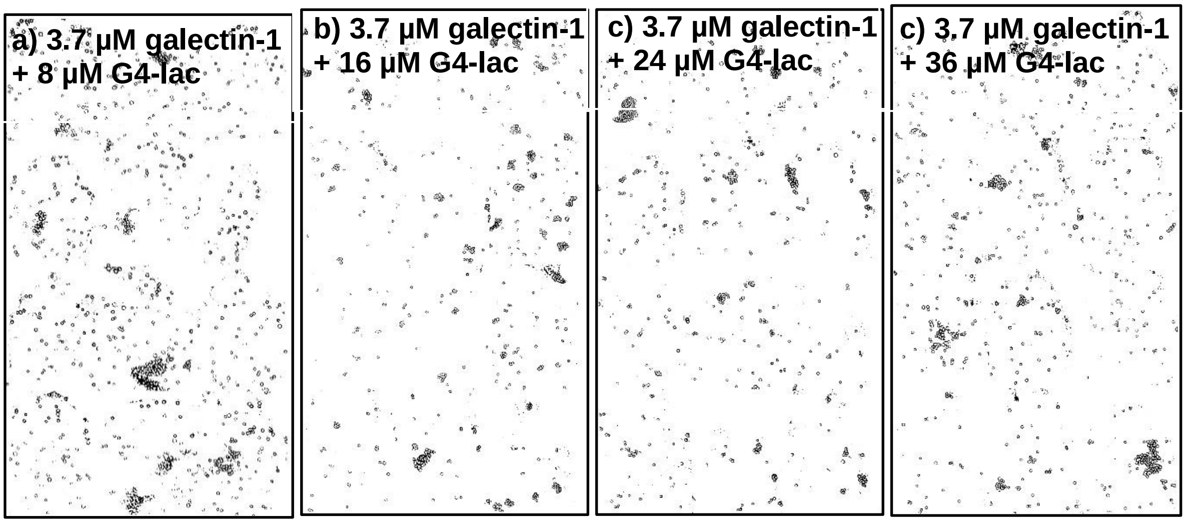

Figure S6: Inhibition of galectin-1 mediated aggregation of DU145 cells by (a) 8 µM **3**, (b) 16 µM **3**, (c) 24 µM **3**, and (d) 36 µM **3**.

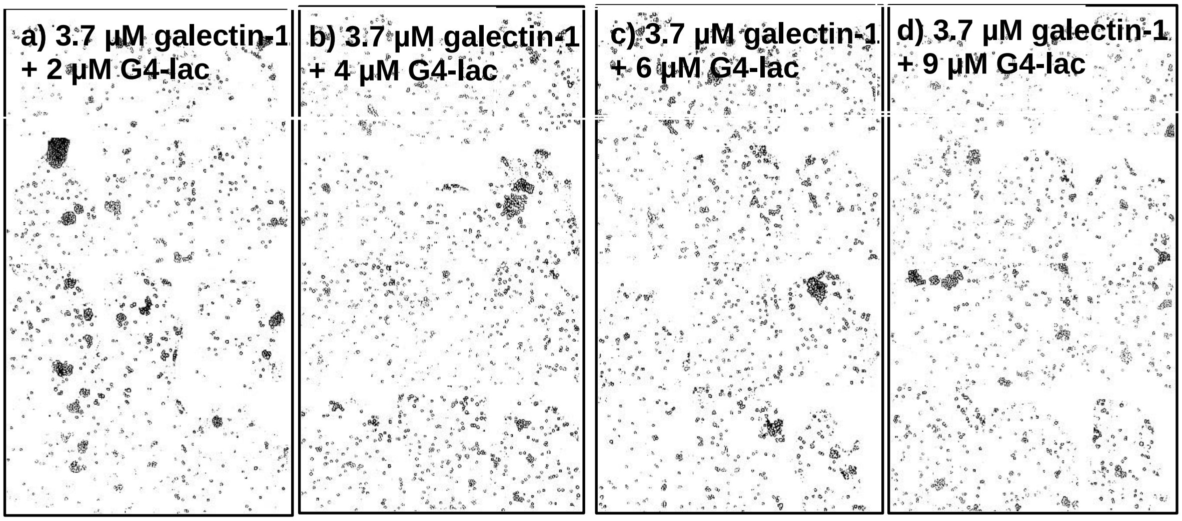

Figure S7: Inhibition of galectin-1 mediated aggregation of DU145 cells by (a) 2 µM **4**, (b) 4 µM **4**, (c) 6 µM **4**, and (d) 9 µM **4**.

Image analysis

Black and white cell aggregation images were analyzed by Pixcavator 5.0. Analysis parameters were set as follows: Size (10); Max Contrast (10); Border Contrast (100); Average Contrast (0); Intensity Dark (0); and, Intensity Light (255). Unmark Light was selected to exclude analysis of light color particles. To quantify cell aggregation, pixel counts corresponding to clumps of 5 or more cells were set as cell aggregates; pixel counts corresponding to fewer than cells were set as free cells. Percent free cells (% free) were determined by dividing the sum of the number of pixels corresponding to free cells by the total number of pixels.

Saturation curve

A saturation curve was prepared using compound **1**. To the DU145 cells (240,000 cells/70 µL) in the presence of galectin-1 (3.7 µM final concentration), **1** was added at a final concentration of 3.3 µM, 16 µM, 33 µM, and 66 µM, respectively. Representative black and white cell images are provided in Figure S8, and the graph shown in Figure S9 reveals a dose responsive inhibition mechanism with **1.**

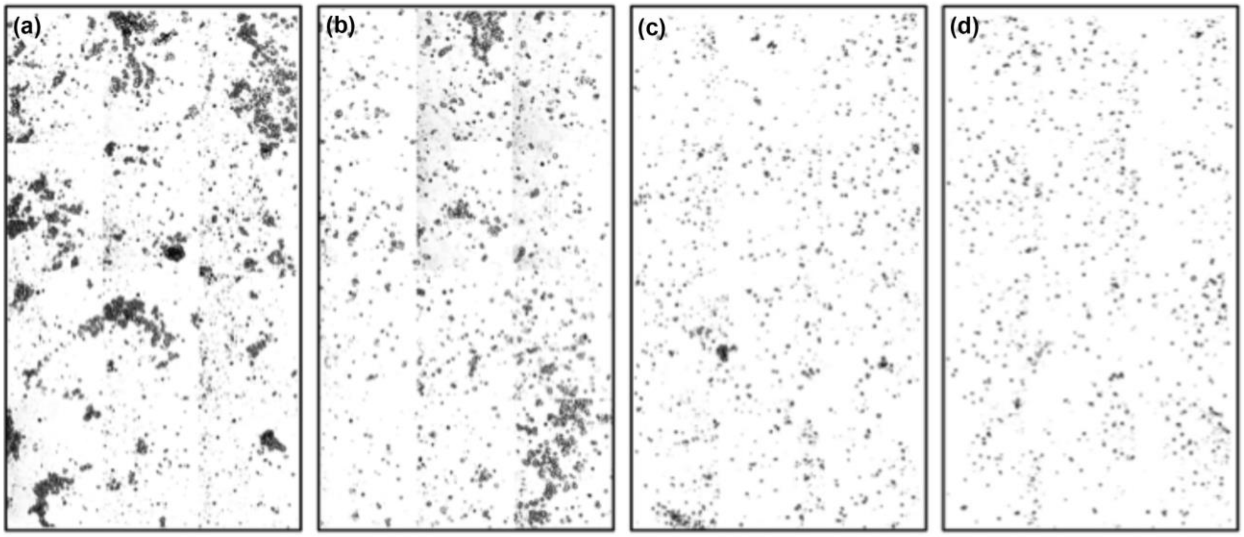

Figure S8: Saturation curve for inhibition of galectin-1 mediated aggregation of DU145 cells. Galectin-1 (3.7 µM) with (a) 3.3 µM **1**, (b) 16 µM **1**, (c) 33 µM **1**, and (d) 66 µM **1**.

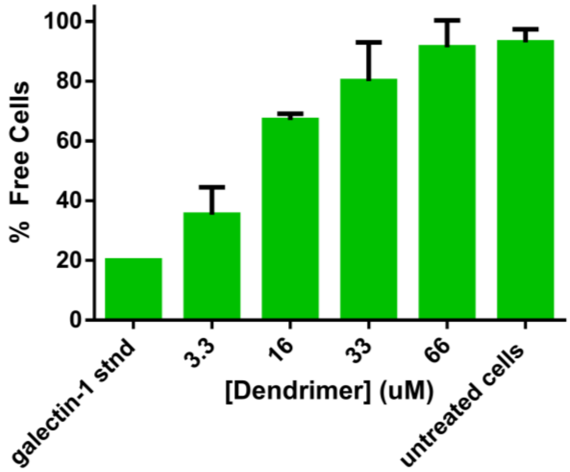

Figure S9: A saturation curve was prepared for the DU145 cells in the presence of 3.7 µM galectin-1 using increasing concentrations of **1**.

Controls

A series of controls were performed to investigate the binding enhancement and the specificity of inhibition using lactose-functionalized dendrimers.

Lactose inhibition

To assess the multivalent enhancement in inhibiting cancer cell adhesion achieved with the dendrimer scaffold, a control assay was performed using monomeric lactoside. Inhibition was observed at 6 mM monomeric lactoside (Figure S10).

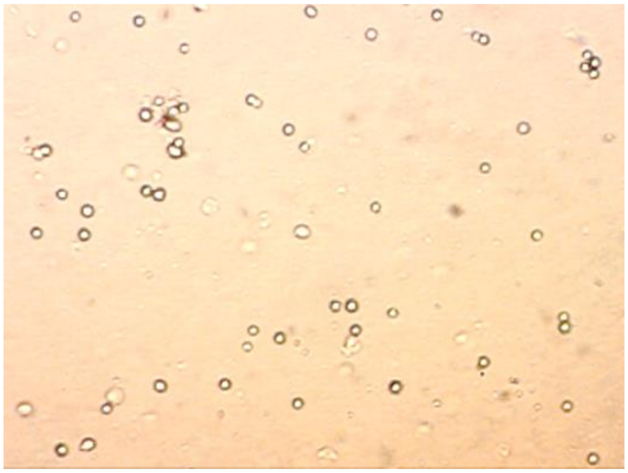

Figure S10: Inhibition of DU145 cellular adhesion by monomeric lactose. Inhibition of cancer cell adhesion by monomeric lactose was observed at 6 mM lactose.

Mannose-functionalized generation 6 PAMAM dendrimer

To investigate the specificity of the lactose-functionalized glycodendrimers, mannose-functionalized generation 6 PAMAM dendrimer (4 µM and 6 µM, final concentrations) was applied to the galectin-1 DU145 system. The mannose functionalized dendrimer did not significantly impact galectin-1 mediated cellular aggregation.

Solution preparation

Assays were run in a final volume of 70 µL. Galectin-1 was prepared at 0.5 mg/mL in PBS buffer (pH 7.4, 15 mM NaCl) from a 7.2 mg/mL stock solution. Glycodendrimer solutions were prepared in millipore H_2O from the lyophilized powder for the following concentrations: (1) 230 µM of **1**; (2) 120 µM of **2**; (3) 56 µM of **3**; and (4) 15 µM of **4**. These glycodendrimer concentrations presented approximately equal concentrations of lactose at the same stages in the cell assay. SFM was prepared by dissolving the DMEM packet (Gibco®) and approximately 1.65 g $NaHCO_3$ in 1000 mL of millipore H_2O. For the DU145 complete media with 10% fetal bovine serum (FBS), to SFM, 5 mL vitamin, 5 mL streptavidin/penicillin mixture, 10 mL essential amino acid, 5 mL non-essential amino acid, and 50 mL FBS were added and filled to 500 mL with SFM and sterilized using a sterile filter unit.

Reference

1. Sun, L.; Crooks, R. M., Dendrimer-mediated immobilization of catalytic nanoparticles on flat, solid supports. *Langmuir* **2002,** *18* (21), 8231-8236.
